# Establishment and validation of an electrocardiogram vector-based machine learning model for the conversion of prone position electrocardiograms into standard electrocardiograms

**DOI:** 10.1093/ehjdh/ztaf146

**Published:** 2025-12-17

**Authors:** Hao Zhang, Zhong-Jian Li, Shi-Feng Li, Xian Shao, Fang-Fang Zhang, Zheng-Kai Xue, Zi-Liang Chen, Jun-Yu Liu, Shen-Da Hong, Shi-Jia Geng, Xu-Hong Geng, Jian-Dong Zhou, Gary Tse, Xing Liu, Hua-Yue Tao, Tong Liu, Kang-Yin Chen

**Affiliations:** Tianjin Key Laboratory of Ionic-Molecular Function of Cardiovascular Disease, Department of Cardiology, Tianjin Institute of Cardiology, Second Hospital of Tianjin Medical University, 23 Pingjiang Road, Hexi District, Tianjin 300211, China; Institute of Electrocardiology of Zhengzhou University, Department of Electrocardiography, The Second Affiliated Hospital of Zhengzhou University, Zhengzhou, China; Institute of Electrocardiology of Zhengzhou University, Department of Electrocardiography, The Second Affiliated Hospital of Zhengzhou University, Zhengzhou, China; Division of Nephrology, National Clinical Research Center for Kidney Disease, State Key Laboratory of Organ Failure Research, Nanfang Hospital, Southern Medical University, Guangzhou, China; Institute of Electrocardiology of Zhengzhou University, Department of Electrocardiography, The Second Affiliated Hospital of Zhengzhou University, Zhengzhou, China; Tianjin Key Laboratory of Ionic-Molecular Function of Cardiovascular Disease, Department of Cardiology, Tianjin Institute of Cardiology, Second Hospital of Tianjin Medical University, 23 Pingjiang Road, Hexi District, Tianjin 300211, China; Tianjin Key Laboratory of Ionic-Molecular Function of Cardiovascular Disease, Department of Cardiology, Tianjin Institute of Cardiology, Second Hospital of Tianjin Medical University, 23 Pingjiang Road, Hexi District, Tianjin 300211, China; Tianjin Key Laboratory of Ionic-Molecular Function of Cardiovascular Disease, Department of Cardiology, Tianjin Institute of Cardiology, Second Hospital of Tianjin Medical University, 23 Pingjiang Road, Hexi District, Tianjin 300211, China; National Institute of Health Data Science and Institute of Medical Technology, Health Science Center, Peking University, Beijing, China; HeartVoice Medical Technology, Hefei, China; Department of Function, Fourth Hospital of Hebei Medical University, Shijiazhuang, China; Department of Family Medicine and Primary Care, Li Ka Shing Faculty of Medicine, The University of Hong Kong, Hong Kong SAR, China; Tianjin Key Laboratory of Ionic-Molecular Function of Cardiovascular Disease, Department of Cardiology, Tianjin Institute of Cardiology, Second Hospital of Tianjin Medical University, 23 Pingjiang Road, Hexi District, Tianjin 300211, China; School of Nursing and Health Studies, Hong Kong Metropolitan University, Hong Kong SAR, China; Tianjin Key Laboratory of Ionic-Molecular Function of Cardiovascular Disease, Department of Cardiology, Tianjin Institute of Cardiology, Second Hospital of Tianjin Medical University, 23 Pingjiang Road, Hexi District, Tianjin 300211, China; Network Information Center, the Second Hospital of Tianjin Medical University, Tianjin, China; Tianjin Key Laboratory of Ionic-Molecular Function of Cardiovascular Disease, Department of Cardiology, Tianjin Institute of Cardiology, Second Hospital of Tianjin Medical University, 23 Pingjiang Road, Hexi District, Tianjin 300211, China; Tianjin Key Laboratory of Ionic-Molecular Function of Cardiovascular Disease, Department of Cardiology, Tianjin Institute of Cardiology, Second Hospital of Tianjin Medical University, 23 Pingjiang Road, Hexi District, Tianjin 300211, China

**Keywords:** Electrocardiogram, Prone ventilation, Prone electrocardiogram, Machine learning, Random forest

## Abstract

**Aims:**

The prone electrocardiogram (ECG) presents challenges in detecting anterior ST-segment elevated myocardial infarction (STEMI). This study aims to develop a method to convert prone ECGs to standard ECGs to facilitate physician diagnosis of STEMI and other cardiovascular diseases (CVD).

**Methods and results:**

The standard ECGs, vectorcardiograms (VCGs), and prone ECGs were prospectively examined for model development. Three conversion approaches were developed: direct lead matching by linear regression (Approach 1), conversion from prone ECGs to VCGs via regression and then to standard ECGs (Approach 2), and machine-learning (ML)-based models (Approach 3). External validation was done with a separate cohort, and a hybrid model was created by integrating the best-performing morphology and amplitude models. The diagnostic performance of the converted ECGs was reviewed by nine cardiologists and benchmarked against the original ECG interpretations. Five hundred and ninety prone ECG cardiac cycles from seventy participants [median age 64 years, interquartile range (IQR) 27.0–70.0] were analysed for model development. The external validation cohort had 94 patients (median age 56.5 years, IQR 39.3–67.0). Approach 3 had the best morphology accuracy, and Approach 2 had the best amplitude similarity. These two models were combined into a hybrid model. In the external validation dataset, the AUCs (95% confidence intervals) for detecting normal ECGs, anterior ST-segment elevation/depression, old anterior myocardial infarction, and bundle branch blocks were 0.835 (0.734–0.908), 0.825 (0.693–0.923), 0.898 (0.799–0.957), 0.867 (0.622–0.956), and 0.910 (0.714–0.953), respectively.

**Conclusion:**

The successful development of models for converting prone ECGs to standard ECGs demonstrated good and robust diagnostic performance for CVD.

## Graphical Abstract

**Graphical Abstract:**
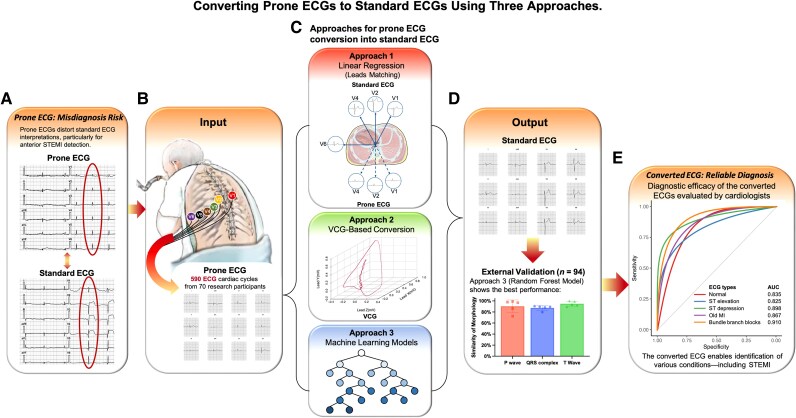
Converting prone electrocardiograms (ECGs) to standard ECGs using three approaches. (*A*) Morphological differences between prone and standard ECG. Prone ECGs show significant morphological differences from standard ECGs, particularly in the precordial leads, making them less reliable for identifying anterior ST-segment elevated myocardial infarction. (*B*) Electrocardiogram leads placement during prone positioning. Leads V1 and V2 are situated on the sides of the seventh thoracic vertebra. Lead V3 is positioned equidistantly between leads V2 and V4. Lead V6 is placed in the fifth intercostal space at the left midaxillary line, and V4 and V5 are aligned with V6 at the same level. (*C*) Three approaches for prone ECG conversion into standard ECG: Approach 1: Utilizing multiple linear regression, the standard ECG leads V1, V2, and V4 were constructed by transforming the prone ECG leads V4, V2, and V1, respectively, into leads that exhibit inverted vectors in the horizontal plane. Approach 2: Utilizing multiple linear regression, a conversion model for prone ECGs and standard vectorcardiograms (VCGs) was established, followed by the application of mature transformation methods to convert them into standard ECGs. Approach 3: Machine learning methods were employed to establish models for converting prone ECG leads into standard ECG. (*D*) Comparisons of the converted standard ECGs and the original ECGs. Approach 3 (random forest model) demonstrated best performance in morphology. The morphology similarity between the converted and original ECGs for P waves, QRS complexes, and T waves in leads V1–V5 was 90.4% (10.1%), 87.4% (3.9%), and 94.9% (4.0%), respectively. (*E*) The diagnostic performance of the converted ECGs was assessed by cardiologists. The receiver operating characteristic (ROC) areas under the curve with 95% confidence intervals (CI) for normal ECGs, ST-segment elevation or depression in anterior leads, old anterior myocardial infarction, and bundle branch blocks were 0.835 (95% CI: 0.734–0.908), 0.825 (95% CI: 0.693–0.923), 0.898 (95% CI: 0.799–0.957), 0.867 (95% CI: 0.622–0.956) and 0.910 (95% CI: 0.714–0.953), respectively.

## Introduction

Since the emergence of coronavirus disease 2019 (COVID-19) in 2019, it has become a global public health crisis, garnering significant international attention.^1^ Coronavirus disease 2019 patients are at risk of developing acute respiratory distress syndrome (ARDS) and multiple organ dysfunction syndrome.^2^ Prone ventilation has been proven to be an essential treatment for ARDS, often requiring patients to remain in the prone position for 12–16 h daily.^3^

Coronavirus disease 2019 and other respiratory viral infections often cause myocardial injury due to hypoxia and hypercoagulability. This is particularly evident in patients requiring invasive mechanical ventilation, who often exhibit abnormal electrocardiograms (ECGs).^4,5^ Consequently, continuous ECG monitoring is imperative for these individuals. However, obtaining accurate ECGs from patients undergoing prone ventilation, especially those on mechanical ventilation, poses challenges. Repositioning these patients typically requires multiple healthcare personnel and can be time-consuming.

Currently, the prevalent method involves recording anterior lead ECGs corresponding to the anterior aspect of the chest in the prone position.^6,7^ While useful for detecting ST-segment/T-wave abnormalities in the limb leads and for rhythm monitoring, prone ECGs show significant morphological differences from standard ECGs, particularly in the precordial leads, making them less reliable for identifying anterior myocardial injury.^7^ Our team previously reported a case of acute anterior wall myocardial infarction in a patient undergoing prone mechanical ventilation. In that case, ST-segment elevation in the anterior wall was nearly overlooked on the prone ECG, leading to delayed detection and prolonged reperfusion time.^8^

Each lead of an ECG is the projection of the vectorcardiogram (VCG) on a specific direction of that lead vector. Prior research has demonstrated the feasibility of converting between ECGs and VCGs.^9,10^ Notably, VCG loops remain consistent regardless of the subject’s position, suggesting that prone ECGs could theoretically be transformed into standard ECGs.

This study aims to develop and validate methods to convert prone ECGs into standard ECGs. Such transformations could aid clinicians in diagnosing critical cardiovascular conditions, including anterior wall ST-segment elevation, and reduce the risk of misdiagnosis and delayed treatment.

## Methods

### Study design and datasets

This two-centre, prospective clinical study utilized two distinct datasets for model development and external validation.

1. Development cohort: The development dataset comprised 70 participants [41 males, 58.6%; median age 64.0 years (IQR: 27.0–70.0)], including 24 healthy volunteers and 46 patients with cardiovascular diseases (CVD), recruited from the Cardiology Department of the Second Hospital of Tianjin Medical University between March and June 2023. A total of 590 prone ECG cardiac cycles were collected. Inclusion criteria were as follows: (i) Healthy volunteers: Normal findings on standard ECG. (ii) Cardiovascular disease (CVD) patients: bundle branch block [complete left bundle branch block (CLBBB), complete right bundle branch block (CRBBB), left anterior hemiblock (LAH)], anterior wall ST-segment elevation [e.g. acute anterior myocardial infarction (MI), anterior wall ventricular aneurysm, Brugada syndrome, or early repolarization syndrome], old anterior MI (without ventricular aneurysm), and anterior wall ST-segment depression. Exclusion criteria included non-sinus rhythm, inability to maintain the prone position for ECG collection, and refusal to provide informed consent.
2. External validation cohort: The external validation dataset included 94 patients [66 males, 70.2%; median age 56.5 years (IQR: 39.3–67.0)] referred for ECG examinations at the outpatient Department of Zhengzhou University Affiliated Second Hospital from June to July 2023. The same inclusion and exclusion criteria as the development cohort were applied. Baseline characteristics of both cohorts are summarized in *Table 1*.

**Table 1:** Demographics data of research participants in two groups

Characteristics	Overall (*n* = 164)	Training dataset (*n* = 56)	Testing dataset (*n* = 14)	External validation set (*n* = 94)	*P*-value
Male, *n* (%)	107 (65.2)	31 (55.4)	10 (71.4)	66 (70.2)	0.165
Age (years)	58.0 (36.0–69.0)	63.0 (27.0–70.8)	66.0 (52.8–72.5)	56.5 (39.3–67.0)	0.401
Height (cm)	168.0 (162.0–174.3)	167.5 (160.0–172.8)	169.0 (162–172.0)	168.0 (165.0–175.0)	0.454
Weight (kg)	67.0 (60.0–75.0)	65.0 (60.0–74.8)	66.5 (59.5–72.5)	68.0 (60.0–75.0)	0.721
BMI (kg/m^2^)	23.66 (21.81–26.15)	23.22 (22.13–26.35)	23.68 (22.17–26.32)	24.09 (21.63–25.95)	0.900
Chest circumference (cm)	90.3 (87.0–94.2)	90.00 (86.75–94.00)	89.50 (83.50–108.75)	91.4 (88.3–94.2)	0.526
Waist circumference (cm)	86.4 (80.5–93)	85.50 (78.75–95.00)	89.00 (79.75–93.25)	86.5 (82.9–92.0)	0.935
ECG classification, *n* (%)					0.389
Normal	56 (34.1)	18 (32.1)	6 (42.9)	32 (34.0)	
ST-segment elevation	28 (17.1)	6 (10.7)	1 (7.1)	21 (22.3)	
Old myocardial infarction	13 (7.9)	7 (12.5)	1 (7.1)	5 (5.3)	
ST-segment depression	22 (13.4)	9 (16.1)	2 (14.3)	11 (11.7)	
Arrhythmia					
CLBBB	5 (3.0)	3 (5.4)	1 (7.1)	1 (1.1)	
CRBBB/RBBB	31 (18.9)	9 (16.1)	2 (14.3)	20 (21.3)	
LAH	9 (5.5)	4 (7.1)	1 (7.1)	4 (4.3)	

Continuous variables are presented as medians [interquartile range (IQR)], and categorical variables as *n* (%). BMI, body mass index; CLBBB, complete left bundle branch block; CRBBB, complete right bundle branch block; ECG, electrocardiogram; LAH, left anterior hemiblock; RBBB, right bundle branch block.

### Data collection

Standard 12-lead ECGs were collected in the supine position, followed by ECG acquisition in the prone position after a rest period of more than 5 min. For prone ECG, leads were positioned as follows: V1 and V2 on either side of the seventh thoracic vertebra, V3 midway between V2 and V4, V6 in the fifth intercostal space at the left midaxillary line, and V4 and V5 at the same horizontal level as V6 (*Graphical Abstract A*). Participants in the development dataset also underwent VCG recordings. Data were acquired using RAGE-12P machines (Nalong Health Technology Co., Ltd., Nanjing, China) at the Second Hospital of Tianjin Medical University and ECG-2350 machines (Nihon Kohden Corporation, Tokyo, Japan) at Zhengzhou University Affiliated Second Hospital. All ECG and VCG recordings were obtained at 500 Hz for 10 s. Age, sex, height, weight, chest circumference, and waist circumference were recorded for all participants.

### Model development

Three distinct methodological approaches were developed to convert prone ECGs into standard ECGs, as summarized in *Graphical Abstract*. Across all models, input features comprised raw signal amplitudes from each of the prone ECG leads, supplemented with key patient characteristics including age, sex, height, weight, chest circumference, and waist circumference.

1. Approach 1 (Linear regression): As illustrated by the horizontal plane projections of the precordial lead vectors in both prone and standard ECG configurations (*Graphical Abstract C*, Approach 1), an inverse vector relationship was identified between specific lead pairs. Specifically, standard ECG lead V1 exhibits an inverse vector relationship with prone ECG lead V4; standard ECG lead V4 inversely corresponds to prone ECG lead V1; and standard ECG lead V2 shows an inverse relationship with prone ECG lead V2. A conversion model was developed for leads V1, V2, and V4 using their inversely correlated prone ECG counterparts (V4, V2, and V1, respectively), derived from healthy volunteers in the development dataset. Demographic variables such as age and sex were also incorporated into the model. Based on both spatial positioning and vector relationships, lead V3 is anatomically situated midway between V2 and V4, while lead V5 is located midway between V4 and V6. Accordingly, leads V3 and V5 were mathematically derived by calculating the mean amplitude values of their respective adjacent leads at each time point using the formulas: derived $\vec{V3}=(\vec{V2}+\vec{V4})/2\;\text{and derived}\;\vec{V5}=(\vec{V4}+\vec{V6})/2$.
2. Approach 2 (ECG–VCG–ECG transformation): Linear regression was used to develop a model combining prone ECGs and standard VCGs. Transformation methods (Dower or Uijen^10^) were applied to convert VCG data into standard ECGs. Transformation coefficients are detailed in Supplementary material online, *Table S1*.
  While the Kors regression matrix has been shown to improve the reconstruction of VCGs from ECGs,^9,11^ its inverse form for VCG to ECG conversion has not been broadly validated. Therefore, we chose the Dower and Uijen methods as representative, clinically accepted matrices for this study.
3. Approach 3 (ML): Using the random forest (RF)^12–14^ and extreme gradient boosting (XGBoost)^15–17^ methods, P-R segments, QRS complexes, and ST-T segments were modelled for prone-to-standard ECG conversion. Data from the development dataset were randomly divided into training and testing datasets (8:2 ratio). To prevent data leakage and ensure generalizability, all ECG cycles from the same participant were exclusively assigned to either the training set or the testing set. This participant-level partitioning guaranteed that no cardiac cycles from the same individual appeared in both sets during model development and evaluation. Random forest regression was performed using the randomForest package (version 4.7-1.1) in R software, with 500 trees (ntree = 500) and default mtry = 6. XGBoost regression was implemented using the xgboost package (version 1.7.5.1) in R software with parameters: eta = 0.1, max_depth = 2, gamma = 0.001, and colsample_bytree = 0.4. The same feature set was used. To further confirm the stability of model performance, we performed five-fold cross-validation within the development dataset. For each fold, performance metrics [root mean squared error (RMSE), mean absolute error (MAE), and coefficient of determination (*R*²)] were calculated, and the mean ± standard deviation across folds was reported. Model hyperparameters (mtry for RF; eta, max_depth, gamma, colsample_bytree, and nrounds for XGBoost) were validated through this cross-validation procedure. After hyperparameter tuning, the final models were retrained on the full training set and evaluated on an independent testing set. The model development and validation process are shown in Supplementary material online, *Figure S1*.

### Model comparison

Model performance was evaluated using RMSE, *R*², and MAE.

### External validation

The models were externally validated by comparing converted ECGs with original standard ECGs in the validation cohort.

1. Amplitude comparison: The amplitudes of P waves, QRS complexes, ST-segments, and T waves (considering bidirectional maximum amplitude) were measured.
2. Morphology comparison: The morphological similarity was evaluated by comparing standardized waveform descriptors—including P-wave morphology (upright, inverted, biphasic, or flat), QRS complex patterns (e.g. qRs, QS, RsR′), and T-wave characteristics (upright, inverted, flat, or biphasic)—across all leads. Morphological consistency was defined as a complete match between the converted ECG and the original standard ECG for each descriptor. The overall morphological similarity was then quantified as the proportion of ECGs in the dataset that exhibited full descriptor-level agreement.

### Hybrid model and diagnostic performance

The hybrid model integrated the optimal morphology and amplitude approaches through scaled combination.

Diagnoses from converted ECGs were assessed by nine independent cardiologists. Discrepancies in diagnoses were resolved by majority consensus. Sensitivity, specificity, and other diagnostic indicators were evaluated for the converted ECGs compared with the original ECG diagnoses.

### Statistical analysis

Statistical analyses were conducted using R software (version 4.2.1, Vienna, Austria). Continuous variables were presented as means ± SD or medians [interquartile range (IQR)] and compared using *t*-tests or Mann–Whitney *U* tests for normally and non-normally distributed continuous variables, respectively. Categorical variables were expressed as frequencies (percentages) and compared using *χ^2^* tests or Fisher's exact tests. Bland–Altman plots were used to assess amplitude agreement between model-predicted and measured amplitudes from the original standard ECG dataset. The significance level was set at *P* < 0.05 for all analyses.

## Results

### Baseline characteristics of participants

A total of 70 participants were included in the development dataset, with a median age of 64.0 (27.0–70.0) years. Among them, 41 (58.6%) were male, including 24 (34.3%) healthy volunteers and 46 (65.7%) CVD patients. Healthy volunteers had significantly lower age, weight, and waist compared with the CVD patients (*P* < 0.05, see Supplementary material online, *Table S2*).

The external validation dataset consisted of 94 patients, with 66 (70.2%) males and a median age of 56.5 (39.3–67.0) years. No significant differences were observed between the development and testing datasets in terms of age, sex, height, weight, chest circumference, or waist circumference. A total of 590 prone ECG cardiac cycles from the 70 participants were included in the final analysis. Furthermore, ECG performance also showed no significant differences between the two datasets (*P* = 0.173). The demographic data of participants in both cohorts are shown in *Table 1*.

### Performance of Approach 1

Using multiple linear regression, V1, V2, and V4 readings of standard ECGs were predicted based on corresponding prone ECG leads. Regression coefficients are presented in Supplementary material online, *Table S3*. The converted leads V4 and V2 demonstrated strong agreement with the corresponding standard ECG leads, achieving *R*² values of 0.804 and 0.729, respectively (both *P* < 0.001), indicating high accuracy. In comparison, the converted lead V1 showed moderate agreement with the standard lead, reflected by an *R*² value of 0.583 (*P* < 0.001).

### Performance of Approach 2

Regression models were used to predict orthogonal leads in standard VCGs. Models incorporating different factors were compared based on the *R*^2^ values for the orthogonal leads, with details shown in Supplementary material online, *Table S4*. The model incorporating prone ECG leads, sex, age, height, and weight achieved high *R*² values of 0.968, 0.968, and 0.845 for orthogonal leads (all *P* < 0.001). The coefficients for this model are presented in *Table 2*.

**Table 2:** The correlation coefficients for the prone electrocardiogram (ECG) to the orthogonal leads of vectorcardiogram (VCG) in Approach 2

	Lead X		Lead Y		Lead Z	
	β coefficients	*P*-value	β coefficients	*P*-value	β coefficients	*P*-value
Lead I	−0.603	0.015	0.730	0.004	−1.413	0.010
Lead II	1.123	<0.001	−1.313	<0.001	0.457	0.473
Lead III	−0.121	0.221	−0.390	<0.001	0.726	0.001
Lead aVR	0.238	0.415	−2.091	<0.001	2.028	0.002
Lead aVL	0.365	<0.001	−0.847	<0.001	1.707	<0.001
Lead aVF	0.045	0.845	0.374	0.108	−0.181	0.722
Lead V1	0.065	<0.001	−0.119	<0.001	0.709	<0.001
Lead V2	0.042	<0.001	0.244	<0.001	−0.397	<0.001
Lead V3	−0.657	<0.001	−0.626	<0.001	2.185	<0.001
Lead V4	0.552	<0.001	−0.181	0.002	−1.317	<0.001
Lead V5	0.055	0.413	−0.191	0.005	1.561	<0.001
Lead V6	0.513	<0.001	0.860	<0.001	−1.166	<0.001
Male	0.067	<0.001	0.134	<0.001	−0.143	0.001
Age	−0.004	0.791	0.333	<0.001	−0.581	<0.001
Height	0.325	<0.001	−0.098	0.130	−0.237	0.094
Weight	−0.394	<0.001	−0.376	<0.001	0.943	<0.001
*R* ^2^	0.968	<0.001	0.968	<0.001	0.845	<0.001

The predicted VCGs were converted into standard ECGs using Dower and Uijen transformations. Morphological and amplitude comparisons were performed between the converted and original standard ECGs: (i) Morphology: For QRS complexes, both transformations produced similar results for leads V1 and V2, while V3 exhibited better morphological similarity with Dower. For V4 and V5, Uijen outperformed Dower (see Supplementary material online, *Figure S2*). (ii) Amplitude: Uijen transformation yielded better overall results for R, S, and T wave amplitudes (see Supplementary material online, *Figure S2*). Based on these findings, a hybrid approach was proposed: Uijen transformation was used for leads V1, V2, V4, and V5, while Dower was preferred for lead V3.

### Performance of Approach 3

Participants in the development dataset were randomly divided into training (80%) and testing (20%) subsets, with no significant demographic differences (see Supplementary material online, *Table S5*).

### Performance of random forest model

**Figure 1:**
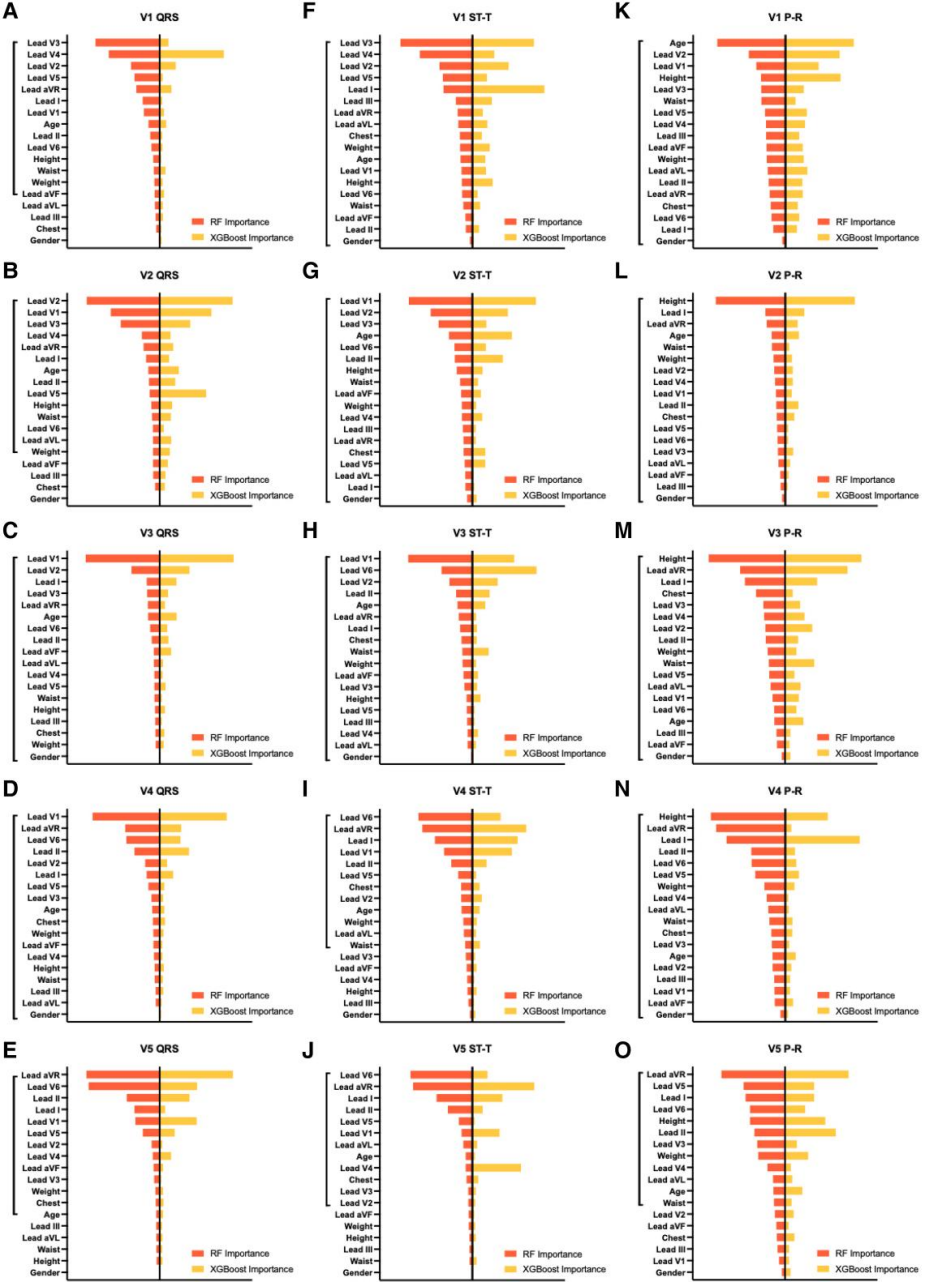
The importance of features for two machine learning (ML) models in different segments (QRS complex, ST-T segment, P-R segment) of leads V1–V5. (*A–E*) Machine learning models for QRS complex of leads V1–V5. (*F–J*) Machine learning models for ST-T segment of leads V1–V5. (*K–O*) Machine learning models for P-R segment of leads V1–V5. The y-axis represents the variables included in the models, with the variables contained in the vertical line indicating those ultimately selected in the random forest (RF) model. The x-axis represents the importance of each variable, where bars on the right indicate the variable importance in the extreme gradient boosting (XGBoost) model, and bars on the left represent the variable importance in the random forest model.

**Figure 2:**
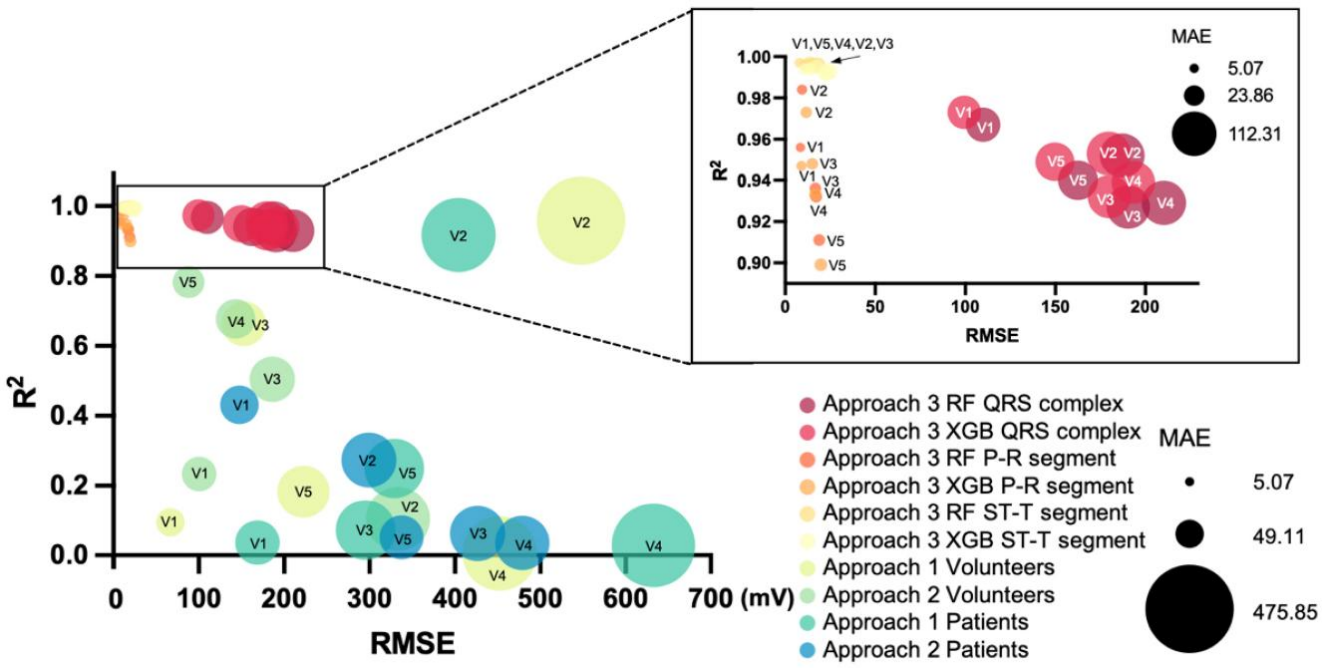
Comparison of evaluation indices for three approaches. The chart illustrates a comparison of evaluation indices for three approaches, with the x-axis representing root mean square error (RMSE), the y-axis representing determination of coefficient [R-squared (*R*^2^)], and marker size indicating mean absolute error (MAE). Models positioned closer to the upper-left corner with smaller markers exhibit better performance. Approaches 1 and 2 calculate model evaluation indices for healthy volunteers and cardiovascular disease, respectively. While Approach 3 calculates evaluation indices for the testing group.

Models were developed for the QRS complex, P-R segment, and ST-T segment. The importance of factors for each lead and segment model is depicted in *Figure 1*. After five-fold cross-validation, the number of variables included in the final models for each lead and segment is shown in the *Figure 1*. In the testing dataset, lead V1 exhibited the best performance in QRS (*R*² = 0.967) and ST-T (*R*² = 0.997) models, while lead V2 performed best for P-R segments (*R*² = 0.984; *Figure 2* and Supplementary material online, *Table S6*).

### Performance of XGBoost model

The importance of each variable in the XGBoost models is also shown in the *Figure 1*. In the testing dataset, the XGBoost model demonstrated similar performance to the RF model, with lead V1 performing best for QRS (*R*² = 0.973) and ST-T segments (*R*² = 0.994). For P-R segments, lead V2 showed the highest *R*² value (*R*² = 0.973, *Figure 2* and Supplementary material online, *Table S6*).

*Figure 2* provides a comparison of evaluation indices between the two ML models. Both models showed comparable performance. RF slightly outperformed in P-R and ST-T models, while XGBoost excelled in the QRS model.

### Cross-validation

Both RF and XGBoost models demonstrated consistent performance across five-fold cross-validation for the QRS complex analysis. For the RF model, average *R*² values (mean ± standard deviation) across leads V1–V5 were 0.935 ± 0.008, 0.914 ± 0.018, 0.915 ± 0.010, 0.928 ± 0.015, and 0.945 ± 0.019, respectively. The XGBoost model achieved comparable performance with *R*² values of 0.942 ± 0.003, 0.912 ± 0.008, 0.906 ± 0.008, 0.932 ± 0.015, and 0.952 ± 0.011 for the corresponding leads. Similar model stability was observed for P-R segment analysis, with RF achieving *R*² values ranging from 0.908 to 0.953 and XGBoost showing performance between 0.857 and 0.940. For ST-T segment assessment, both models exhibited excellent performance, with RF maintaining *R*² values exceeding 0.990 and XGBoost achieving values between 0.975 and 0.983.

These cross-validation results confirm the robustness of both modelling approaches and are consistent with the performance metrics obtained from the single training/test split reported in Supplementary material online, *Table S7*, thereby validating the generalizability and stability of our models.

### Comparison evaluation indices of the three approaches

Evaluation indices for the three approaches are summarized in *Figure 2* and Supplementary material online, *Table S6*. Both Approach 1 and Approach 2 demonstrated lower accuracy than the ML models, regardless of whether applied to healthy volunteers or CVD patients.

### External validation of the three models

**Figure 3:**
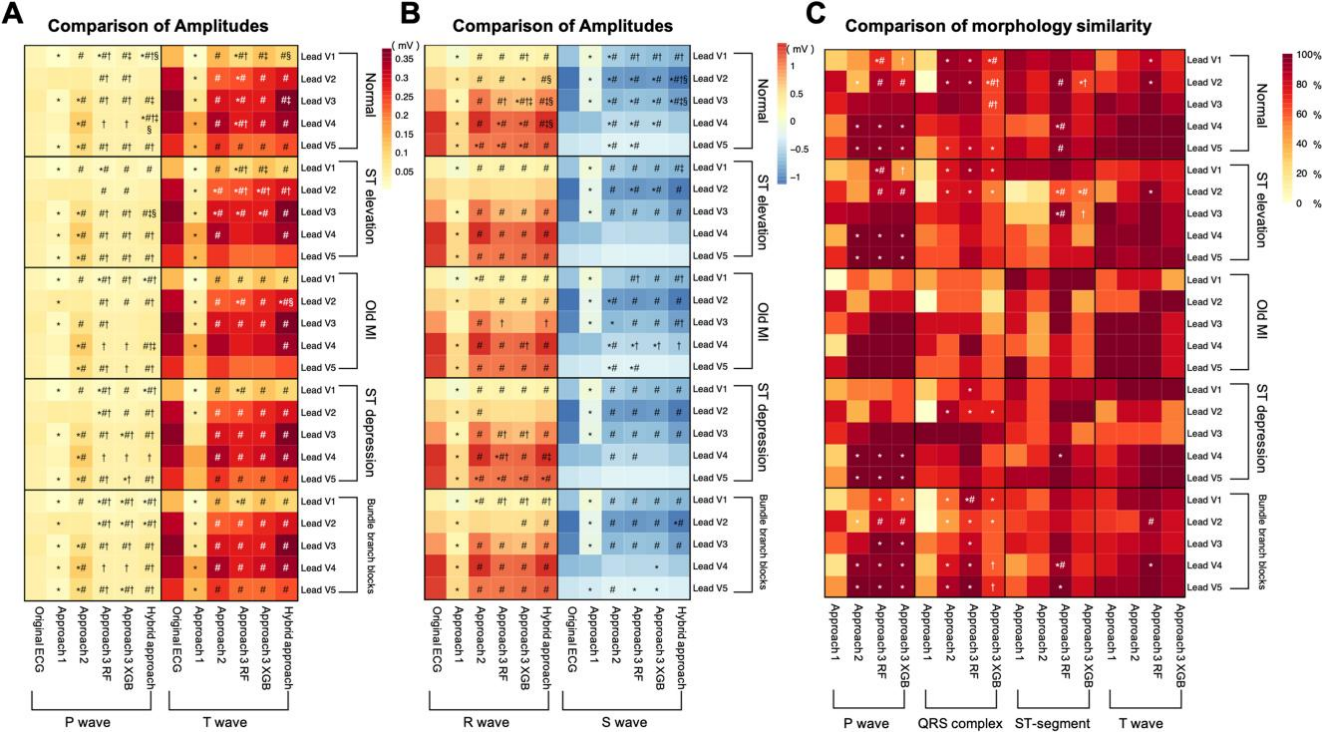
Comparison of amplitudes and morphology in electrocardiograms (ECGs) of external validation dataset after transformation using each approach and comparison with the original electrocardiograms. (*A*) Comparison of average amplitudes of P waves and T waves in converted electrocardiograms with the original electrocardiograms. (*B*) Comparison of average amplitudes of R waves and S waves in converted electrocardiograms with the original electrocardiograms. In (*A* and *B*), *, indicates a significant difference in amplitude compared to the original electrocardiogram (*P* < 0.05), # indicates a significant difference in amplitude compared to the electrocardiogram converted using the Approach 1 (*P* < 0.05), † indicates a significant difference in amplitude compared to the electrocardiogram converted using the Approach 2 (*P* < 0.05), ‡ indicates a significant difference in amplitude compared to the electrocardiogram converted using the Approach 3 (the RF method) (*P* < 0.05), and § indicates a significant difference in amplitude compared to the electrocardiogram converted using the Approach 3 (XGBoost method) (*P* < 0.05). (*C*) Comparison of morphology similarity in each segment of converted electrocardiograms with the original electrocardiograms. *, indicates a significant difference in morphology similarity compared to Approach 1 (*P* < 0.05), #, indicates a significant difference in morphology similarity compared to Approach 2 (*P* < 0.05), †, indicates a significant difference in morphology similarity compared to Approach 3 (the RF method) (*P* < 0.05). MI, myocardial infarction; ST elevation, ST-segment elevation; ST depression, ST-segment depression; RF, random forest model; XGB, eXtreme Gradient Boosting (XGBoost) model.

Three approaches were applied to transform the external validation ECG data, and the converted ECGs were compared with the original standard ECGs. (i) Amplitude: Approach 1 exhibited decreased amplitudes across all segments compared to the original ECGs. Approach 2 produced closer amplitudes, except for an increased P wave. Both ML-based methods (Approach 3) showed the slightly lower mean values compared with original ECGs (*Figure 3A* and *B*). (ii) Morphology: Among all approaches, Approach 3 (RF models) achieved the highest morphological similarity. The average morphological similarity across leads for the P wave, QRS complex, ST segment, and T wave were 90.4 ± 10.1%, 87.5 ± 3.9%, 91.7 ± 3.2%, and 94.9 ± 4.0%, respectively (*Figure 3C*).

### Development and validation of hybrid models

**Figure 4:**
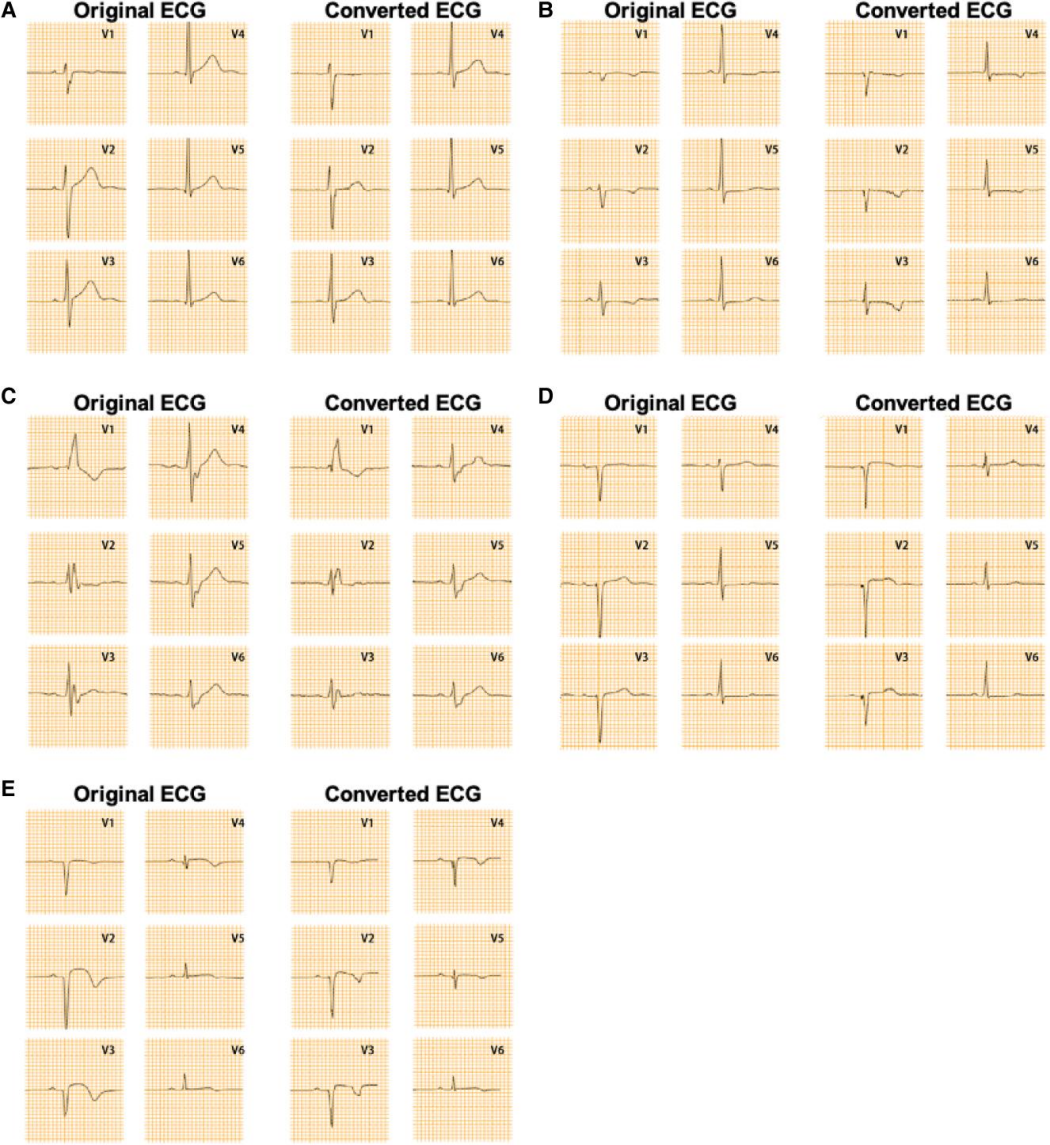
Comparison of electrocardiograms (ECGs) converted from different types of prone electrocardiograms using hybrid models in the external validation set with the original standard electrocardiograms. (*A*) Normal electrocardiogram; (*B*) ST-segment depression; (C) Bundle branch block (complete right bundle branch block); (*D*) old anterior wall myocardial infarction; (*E*) ST-segment elevation.

To leverage the strengths of both amplitude and morphological predictions, Approach 3 (RF model) was adjusted in amplitude based on Approach 2, which demonstrated the best amplitude similarity. This resulted in the development of a hybrid model. Examples of ECGs converted using the hybrid models in the external validation dataset are provided in *Figure 4*.

The correlation of amplitudes and Bland–Altman analyses for each lead and waveform segment of the hybrid model in the external validation dataset are shown in Supplementary material online, *Figure S3*. The results revealed the highest agreement for lead V5, with *R*² values of 0.798, 0.597, and 0.756 for the R wave, S wave, and T wave, respectively (*P* < 0.001).

### Diagnostic performance of hybrid models

**Figure 5:**
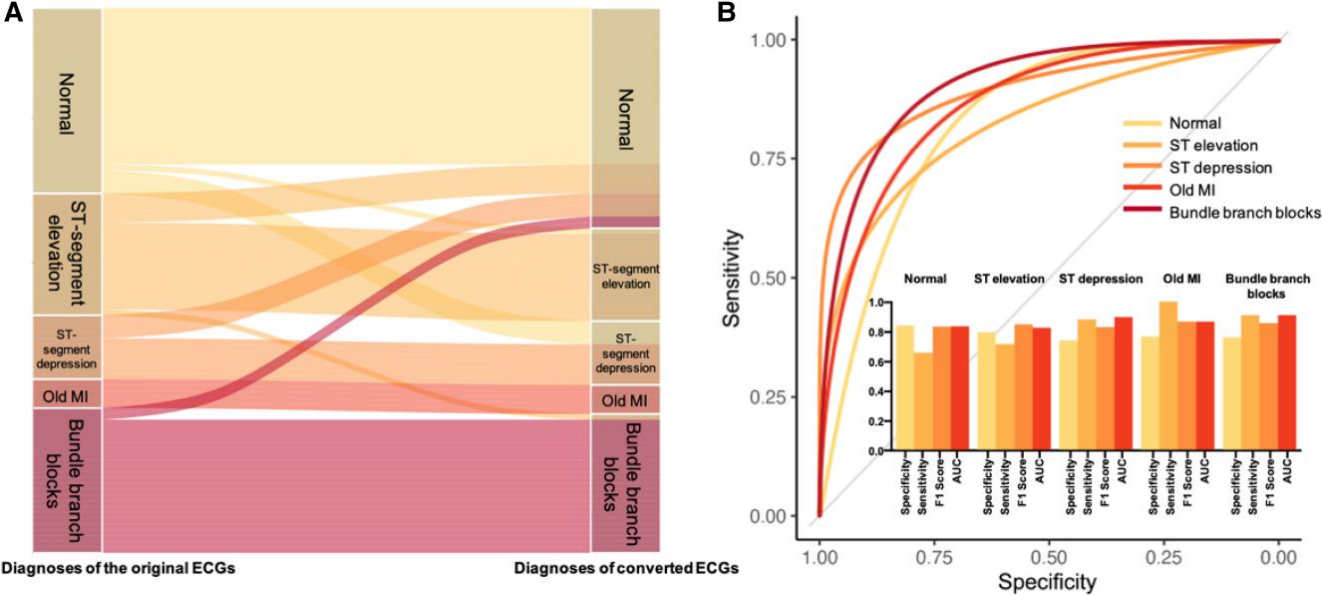
The diagnoses comparison between original electrocardiograms (ECGs) and converted standard electrocardiograms diagnosed by experts in the external validation dataset (*A*) and the predictive performance of the converted standard electrocardiograms for each type of electrocardiograms (*B*). (*A*) The left y-axis represents the diagnoses of each original electrocardiogram in the external validation dataset, while the right y-axis shows the diagnoses provided by experts for the electrocardiograms converted through the model. (*B*) Receiver operating characteristic (ROC) curves for the diagnoses of the conversed electrocardiograms based on expert assessments are displayed. The bar graph illustrates the sensitivity, specificity, area under the receiver operating characteristic curve (AUC), and F1 score of the models for various disease. MI, myocardial infarction.

Comparisons of diagnoses made with the original ECGs vs. those made with the converted ECGs for the training and testing dataset are shown in Supplementary material online, *Table S8*. In the training set, the diagnostic performance was favourable, with areas under the curve (AUCs) of 0.925 [95% confidence intervals (CI): 0.897–0.953] for normal ECGs, 0.948 (95% CI: 0.910–0.985) for ST-segment elevation, 0.909 (95% CI: 0.873–0.946) for ST-segment depression, 0.966 (95% CI: 0.933–0.998) for old MI, and 0.992 (95% CI: 0.982–1.000) for bundle branch blocks. In the testing dataset, the diagnostic performance was comparable, except for ST-segment depression (AUC = 0.700, 95% CI: 0.540–0.860). In the external validation dataset, the hybrid model demonstrated excellent diagnostic performance, especially for bundle branch blocks, with an AUC reaching 0.910 and an F1 score reaching 0.857. It also showed good diagnostic efficacy for diagnosing ST-segment elevation and depression, with AUCs of 0.825 (95% CI: 0.693–0.923) and 0.898 (95% CI: 0.799–0.957), respectively (*Figure 5*).

### Subgroup analysis

Subgroup analysis of ECG performance in the external validation dataset is presented in *Figure 3*. Regarding amplitude, the hybrid model showed strong correlations with the original ECGs across all subgroups (*Figure 3A, B*). In terms of morphological similarity, Approach 3 (RF) consistently exhibited the highest morphological similarity across all subgroups. The similarity was particularly high in the subgroup of patients with old MI, where the converted ECGs from all models showed no statistical differences (*Figure 3C*).

When diagnostic performance was evaluated, overall accuracy was maintained across subgroups, though moderate variations were observed across subgroups (see Supplementary material online, *Table S9* and Supplementary material online, *Figure S5*). By sex, performance was higher in males (AUC 0.875, 95% CI 0.801–0.923; sensitivity 84.7%) than in females (AUC 0.704, 95% CI 0.455–0.914; sensitivity 66.7%), while specificity exceeded 91% in both groups. Age-stratified analysis showed comparable results, with AUCs of 0.875 (95% CI 0.794–0.933) in participants >50 years and 0.832 (95% CI 0.704–0.919) in those ≤50 years. The clearest separation was seen with body mass index (BMI): patients with BMI >24 kg/m^2^ achieved excellent performance (AUC 0.973, 95% CI 0.926–0.993; sensitivity 93.8%; specificity 98.4%), whereas those with BMI ≤ 24 kg/m^2^ had lower sensitivity (71.7%) and reduced AUC (0.786, 95% CI: 0.674–0.868).

Taken together, these findings indicate that the proposed model maintains robust performance across clinically relevant subgroups. Diagnostic accuracy was consistently high in overweight individuals, while sensitivity was somewhat attenuated in females and leaner participants, highlighting potential areas for future model optimization.

### Model interpretability analysis

To enhance model interpretability, we conducted Shapley Additive Explanations (SHAP) analysis, which quantifies the contribution of each feature to the model's predictions through SHAP values.^18^ These values not only reflect the magnitude of each feature's impact on prediction performance but also reveal whether the relationship is positive or negative. As shown in Supplementary material online, *Figure S4*, the SHAP analysis of our best-performing model (RF) across leads V1–V5 provides a comprehensive visualization of feature importance (ranked in descending order) and clearly illustrates the directional relationships between ECG waveform characteristics and their corresponding clinical outcomes.

## Discussion

This study developed three approaches to convert prone ECGs into standard ECGs and evaluated their performance using an external validation dataset. A hybrid model was constructed by integrating the strengths of these approaches, demonstrating high diagnostic accuracy for CVDs, including anterior wall old MI, ST-segment elevation, and ST-segment depression.

To our knowledge, this study is the first to utilize ML algorithms and electrocardiographic vector principles for transforming prone ECGs into standard ECGs. This novel approach allows clinicians to obtain standard ECGs without repositioning patients, thereby enhancing diagnostic accuracy and reducing the risk of misdiagnosis or delayed recognition life-threatening conditions, such as anterior wall ST-segment elevation MI.

The prevalence of COVID-19 and other respiratory viruses has led to increased attention on prone ventilation. Additionally, impaired pulmonary function and chronic pulmonary diseases are recognized as independent risk factors for CVD.^19^ This highlights the critical importance of continuous ECG monitoring in patients undergoing prone ventilation, especially those with comorbid conditions such as coronary artery disease and heart failure. However, these patients often require mechanical ventilation, making it difficult to adjust their body position for standard ECG acquisition.

Consequently, researchers have proposed several solutions to address this issue: (i) Pre-positioning chest lead electrodes: Placing electrodes on the chest before assuming the prone position produces ECG waveforms highly similar to standard ECGs. However, prolonged placement may lead to adverse effects, such as pressure sores.^20^ (ii) Mirror-image vector connections: Utilizing chest lead electrodes on the back with a mirror-image vector connection generates ECGs resembling standard waveforms,^21^ enabling the diagnosis of conditions such as bundle branch block.^22^ However, this method reduces ECG amplitude due to the greater distance between electrodes and the heart, which may obscure critical information, such as ST-segment deviations. Additionally, electrode misplacement poses a significant risk, potentially leading to misdiagnoses.^23^ (iii) Standard mirror-connection method: This approach, also employed in this study, is the most commonly used method for acquiring prone ECGs due to its convenience. However, its significant waveform differences from standard ECGs remain a major limitation.

In this study, Approaches 1 and 2 utilized electrocardiographic vector concepts, originally proposed by Burger *et al*.^24^ in 1952, to transform VCGs into standard ECGs. Subsequently, many researchers have explored this issue.^25–27^ Among all the models, Approach 2 achieved better performance, producing converted ECGs with amplitudes closely resembling those of standard ECGs.

With the rapid development of ML technology, these methods have increasingly been applied to ECG and VCG transformations.^28^ However, no prior studies have explored ML-based transformations between prone and standard ECGs. Building upon previous research, this study introduced an ML-based Approach 3 that leveraged features imperceptible to the human eye,^29^ significantly improving the accuracy of the converted ECGs. The model development used two ML algorithms, RF and XGBoost, which are characterized by efficiency and accuracy, and are widely applied to regression tasks.^12,17,30–32^ Results demonstrated that Approach 3 outperformed the other methods, producing ECGs with the highest morphological similarity to the original standard ECGs.

The diagnostic efficacy of the hybrid model was further validated externally, showing excellent performance in identifying old MI and bundle branch block. These conditions exhibit substantial morphological differences from normal ECGs, making them easier to distinguish. However, diagnostic performance for ST-segment elevation and depression was slightly lower. This may be attributed to the smaller sample size and minimal morphological differences between these ECG types and normal ECGs, which rely primarily on amplitude changes for differentiation. This limitation can be addressed by increasing the sample size to improve the predictive performance of the model, or by introducing deep learning models, which might improve prediction accuracy.^33^

Furthermore, beyond the regression models employed in this study, recent advancements in generative artificial intelligence present a promising alternative avenue for ECG signal transformation. For instance, Alcaraz and Strodthoff^34^ recently proposed SSSD-ECG, a diffusion model combined with structured state space models, which successfully generates realistic 12-lead ECGs conditioned on a wide array of over 70 clinical statements. Their work, which includes a clinical Turing test demonstrating that experts often cannot distinguish generated ECGs from real ones, highlights the immense potential of such generative approaches in capturing the complex underlying distribution of ECG signals.

While our current study focuses on converting existing prone ECGs into their standard supine counterparts using regression and machine learning techniques, the ultimate goal aligns with that of generative models: to produce standard-form ECGs that are clinically reliable. Future research could explore adapting these powerful generative architectures, such as conditional diffusion models, for the specific task of cross-posture ECG translation. Instead of learning a direct mapping from prone to supine signals, a generative model could learn to produce a plausible standard ECG given a prone ECG input and potentially other patient information.

### Limitations

This study is the first to propose and validate methods for converting prone ECGs into standard ECGs, enabling acquisition without altering patient position, which is particularly valuable in critical care settings. However, the study has limitations. First, although the data were prospectively collected, the sample size of both the development and external validation cohorts, while adequate for a proof-of-concept investigation, remains limited. Future studies involving larger, multi-centre, and more diverse populations are warranted to enhance the generalizability and robustness of our findings. Second, the study cohort consisted exclusively of Asian participants in sinus rhythm, which may limit the extrapolation of results to other demographic groups or patients with arrhythmias. The relatively small and homogeneous development dataset may have introduced potential bias, although the models showed satisfactory performance during external validation. Furthermore, the current models incorporated general patient anthropometric data. While such measures are typically available in clinical settings and physiologically relevant, they must be accurately recorded to ensure optimal model performance. In certain scenarios—such as critical care where rapid or approximate measurements are taken—data inaccuracies could affect model applicability. Future research should expand the study population, increase the dataset size, and explore models based solely on ECG data to improve generalizability and robustness.

## Conclusions

This study introduces a hybrid model that combines the strengths of ML and ECG vector-based approaches. In external validation, the converted ECGs exhibited high diagnostic accuracy for both normal ECGs and CVDs. This innovative method offers a practical solution for monitoring ECGs in patients who must remain in the prone position. This technique could benefit patients who cannot assume a supine position due to respiratory distress, spinal injuries, or during specific surgical procedures.

## Supplementary Material

ztaf146_Supplementary_Data

## Data Availability

Data are available by contacting the corresponding author on reasonable requests.
