# Supplementary material for: Establishment and validation of an electrocardiogram vector-based machine learning model for the conversion of prone position electrocardiograms into standard electrocardiograms
Source: Eur Heart J Digit Health. 2025 Dec 17;7(3):ztaf146. doi: 10.1093/ehjdh/ztaf146 (PMC12994467; doi:10.1093/ehjdh/ztaf146)
Supplement: ztaf146_Supplementary_Data [file ztaf146_supplementary_data.docx]

**Supplementary Information**

***Establishment and Validation of an ECG Vectors-Based Machine Learning Model for the Conversion of Prone Position ECGs into Standard ECGs***

**Table S1.** The coefficients of Dower and Uijen transformation.

**Table S2.** Demographics data of development dataset.

**Table S3** The Coefficients of Approach 1

**Table S4.** Coefficients for each orthogonal lead in different models in Approach 2.

**Table S5.** Demographics data and electrocardiogram (ECG) parameters of training and testing dataset.

**Table S6.** The comparison of model performance for each approach.

**Table S7.** Comparison of the model's diagnostic performance in training, testing, and external validation datasets

**Table S8.** Subgroup performance of the model's diagnostic performance in the external validation datasets

**Figure S1.** The framework of the model development and validation.

**Figure S2.** The comparison of morphology and amplitude between the converted standard electrocardiograms (ECGs) using Approach 2 and the original ECGs.

**Figure S3.** Correlation of amplitudes and Bland-Altman analysis for each lead and waveform segment of electrocardiograms (ECGs) converted by the hybrid model in external validation set.

**Figure S4.** Shapley additive explanation (SHAP) values for features the random forest model (Approach 3) to predict different segments (QRS complex, ST-T segment, P-R segment) of leads V1-V5.

**Figure S5.** Areas under the receiver operating characteristic curve (AUROC) and 95% confidence interval (CI) of model diagnosis for each subgroup in the external validation dataset.

**Table S1.** **The coefficients of Dower and Uijen transformation**

| Lead | Dower transformation | | | Uijen transformation | | |
| --- | --- | --- | --- | --- | --- | --- |
|  | Lead X | Lead Y | Lead Z | Lead X | Lead Y | Lead Z |
| I | 0.632 | -0.235 | 0.059 | 0.79 | -0.24 | 0.08 |
| II | 0.235 | 1.066 | -0.132 | 0.24 | 0.105 | -0.01 |
| III | -3.970 | 1.301 | -0.191 | -0.56 | 1.29 | 0.09 |
| aVR | -0.434 | -0.415 | 0.037 | -0.51 | -0.41 | -0.03 |
| aVL | 0.515 | -7.680 | 0.125 | 0.67 | -0.77 | 0.08 |
| aVF | -0.081 | 1.184 | -0.162 | -0.16 | 1.17 | -0.05 |
| V1 | -0.515 | 0.157 | -9.170 | -0.52 | -0.06 | -1.04 |
| V2 | 0.044 | 0.164 | -1.390 | -0.15 | -0.35 | -1.76 |
| V3 | 0.882 | 0.098 | -1.280 | 0.69 | 0.38 | 1.16 |
| V4 | 1.213 | 0.127 | -0.601 | 1.34 | 0.68 | -0.49 |
| V5 | 1.125 | 0.127 | -0.860 | 1.09 | 0.64 | 0.01 |
| V6 | 0.831 | 0.076 | 0.230 | 0.65 | 0.52 | 0.23 |

**Table S2. Demographics data of development dataset**

|  | Overall  （*n* = 70） | Healthy volunteers  (*n* = 24) | Cardiovascular disease patients  (*n* = 46) | *P* value |
| --- | --- | --- | --- | --- |
| Age (years) | 64.0 (27.0-70.0) | 26.0 (24.0-45.0) | 68.0 (58.0-74.0) | <0.001 |
| Male, *n* (%) | 41(58.6) | 13(54.2) | 28(60.9) | 0.618 |
| Height, (cm) | 166.2±8.5 | 166.1±7.8 | 166.3±9.0 | 0.934 |
| Weight, (kg) | 65.0 (60.0-74.25) | 62.5 (51.5-70.0) | 67.5 (62.0-80.0) | 0.020 |
| Body mass index, (kg/m^2^) | 23.66 (21.81-26.15) | 23.23 (22.44-27.12) | 23.22 (22.17-25.33) | 0.310 |
| Chest circumference, (cm) | 90.0 (85.0-96.0) | 88.0 (83.0-91.75) | 90.0 (87.0-99.0) | 0.066 |
| Waist circumference, (cm) | 86.9±12.5 | 79.4±9.6 | 91.1±12.0 | <0.001 |
| ECG classification, *n* (%) |  |  |  | <0.001 |
| ST-segment elevation | 7 (10.0) | 0 (0.0) | 7 (15.2) |  |
| Old myocardial infarction | 8 (11.4) | 0 (0.0) | 8 (17.4) |  |
| ST-segment depression | 11 (15.7) | 0 (0.0) | 11 (23.9) |  |
| Bundle branch block |  |  |  |  |
| CLBBB | 4 ( 5.7) | 0 (0.0) | 4 ( 8.7) |  |
| CRBBB/RBBB | 11 (15.7) | 0 (0.0) | 11 (23.9) |  |
| CRBBB+LAH | 2 ( 2.9) | 0 (0.0) | 2 ( 4.3) |  |
| LAH | 3 ( 4.3) | 0 (0.0) | 3 ( 6.5) |  |

Data are *n* (%) for categorical variables and median (IQR) or mean ± standard deviation for continuous variables.

CLBBB, complete left bundle branch block; CRBBB, complete right bundle branch block; ECG, electrocardiogram; IQR: interquartile range; LAH, left anterior hemiblock; RBBB, right bundle branch block.

**Table S3. The Coefficients of Approach 1**

|  | Lead V2 | | Lead V4 | | Lead V1 | |
| --- | --- | --- | --- | --- | --- | --- |
|  | β coefficients | P value | β coefficients | P value | β coefficients | P value |
| Corresponding prone ECG lead | (Prone V2)  0.136 | <0.001 | (Prone V1)  -0.591 | <0.001 | (Prone V4)  -0.140 | <0.001 |
| Male | 0.466 | <0.001 | 0.005 | 0.804 | 0.622 | <0.001 |
| Age | -0.417 | <0.001 | -0.225 | <0.001 | 0.326 | <0.001 |
| Height | 0.047 | 0.622 | 0.597 | <0.001 | 0.298 | 0.016 |
| Weight | -1.169 | <0.001 | 0.577 | <0.001 | -2.283 | <0.001 |
| Chest circumference | -0.376 | 0.005 | -1.201 | <0.001 | 0.997 | <0.001 |
| Waist circumference | 2.244 | <0.001 | 0.587 | <0.001 | 0.978 | <0.001 |
| R^2^ | 0.729 | <0.001 | 0.804 | <0.001 | 0.583 | <0.001 |

ECG, electrocardiogram.

**Table S4. Coefficients for each orthogonal lead in different models in Approach 2**

| Variables included in the model | Lead X | Lead Y | Lead Z |
| --- | --- | --- | --- |
| ECG | 0.967 | 0.961 | 0.825 |
| ECG + Gender + Age | 0.967 | 0.951 | 0.787 |
| ECG + Gender + Age + Height + Weight | 0.968 | 0.968 | 0.845 |
| ECG + Gender + Age + Height + Weight + Chest and Waist circumference | 0.973 | 0.976 | 0.852 |

ECG, electrocardiogram.

**Table S5. Demographics data and electrocardiogram (ECG) parameters of training and testing dataset**

| Characteristics | Training Dataset  (*n* = 56) | Testing Dataset  (*n*= 14) | *P* value |
| --- | --- | --- | --- |
| Male, *n* (%) | 31 (55.4) | 10 (71.4) | 0.368 |
| Age, (years) | 63.0 (27.0-70.75) | 66.0 (52.75-72.5) | 0.597 |
| Height, (cm) | 166.00±8.79 | 167.21±7.45 | 0.636 |
| Weight, (kg) | 65.0 (60.0-74.75) | 66.5 (59.5-72.5) | 0.477 |
| BMI, (kg/m^2^) | 23.22 (22.13-26.35) | 23.68 (22.17-26.32) | 0.860 |
| Chest circumference, (cm) | 90.0 (86.75-94.0) | 89.5 (83.5-108.75) | 0.638 |
| Waist circumference, (cm) | 85.5 (78.75-95.0) | 89.0 (79.75-93.25) | 0.735 |
| ECG classification, *n* (%) |  |  | 0.997 |
| Normal | 18 (32.1) | 6 (42.9) |  |
| Abnormal | 38 (67.9) | 8 (57.1) |  |
| ST-segment elevation | 6 (10.7) | 1 ( 7.1) |  |
| Old myocardial infarction | 7 (12.5) | 1 ( 7.1) |  |
| ST-segment depression | 9 (16.1) | 2 (14.3) |  |
| Bundle branch block |  |  |  |
| CLBBB | 3 ( 5.4) | 1 ( 7.1) |  |
| CRBBB/RBBB | 9 (16.1) | 2 (14.3) |  |
| LAH | 4 ( 7.1) | 1 ( 7.1) |  |
| ECG cardiac cycles | 470 | 120 |  |
| ECG characteristics |  |  |  |
| P wave duration, (ms) | 111.0 (100.0-128.5) | 108.0 (98.5-123.0) | 0.419 |
| P-R duration, (ms) | 180.0 (150.0-197.0) | 173.0 (152.5-185.5) | 0.494 |
| QRS complex, (ms) | 96.0 (90.0-108.0) | 100.0 (89.5-129.5) | 0.471 |
| QT duration, (ms) | 406.0 (382.0-450.5) | 421.0 (381.0-455.0) | 0.901 |
| QTc duration, (ms) | 427.0 (411.5-450.0) | 438.0 (406.5-451.75) | 0.849 |
| Heart rate, (bpm) | 69.0 (64.0-76.0) | 66.0 (58.25-77.25) | 0.670 |
| RV5, (mV) | 1.20 (0.67-1.55) | 0.99 (0.68-1.09) | 0.150 |
| SV1, (mV) | 0.69 (0.37-1.00) | 0.67 (0.33-1.30) | 0.971 |

Data are *n* (%) for categorical variables and median (IQR) or mean ± standard deviation for continuous variables.

BMI, body mass index; CLBBB, complete left bundle branch block; CRBBB, complete right bundle branch block; LAH, left anterior hemiblock; RBBB, right bundle branch block; RV5, Amplitude of R wave in lead V5; SV1, Amplitude of S wave in lead V1.

**Table S6. The comparison of model performance for each approach**

|  | Lead V1 | Lead V2 | Lead V3 | Lead V4 | Lead V5 |
| --- | --- | --- | --- | --- | --- |
| Approach 1 |  |  |  |  |  |
| Volunteers |  |  |  |  |  |
| RMSE, (mV) | 66.594 | 547.463 | 152.510 | 452.234 | 222.384 |
| R^2^ | 0.094 | 0.958 | 0.663 | 0.005 | 0.182 |
| MAE, (mV) | 49.214 | 475.853 | 122.383 | 338.572 | 166.367 |
| Patients |  |  |  |  |  |
| RMSE, (mV) | 168.585 | 404.103 | 295.229 | 632.518 | 328.872 |
| R^2^ | 0.036 | 0.917 | 0.072 | 0.028 | 0.249 |
| MAE, (mV) | 120.584 | 336.403 | 218.576 | 427.576 | 216.200 |
| Approach 2 |  |  |  |  |  |
| Volunteers |  |  |  |  |  |
| RMSE, (mV) | 100.215 | 333.123 | 185.994 | 142.979 | 87.842 |
| R^2^ | 0.233 | 0.104 | 0.504 | 0.677 | 0.782 |
| MAE, (mV) | 73.221 | 251.298 | 126.463 | 95.388 | 62.061 |
| Patients |  |  |  |  |  |
| RMSE, (mV) | 147.440 | 299.102 | 426.389 | 478.766 | 337.324 |
| R^2^ | 0.431 | 0.273 | 0.062 | 0.035 | 0.051 |
| MAE, (mV) | 90.767 | 184.076 | 186.664 | 180.366 | 117.682 |
| Approach 3 (Random Forest model) | |  |  |  |  |
| QRS complex |  |  |  |  |  |
| Training dataset |  |  |  |  |  |
| RMSE, (mV) | 59.177 | 89.341 | 72.285 | 65.205 | 50.384 |
| R^2^ | 0.991 | 0.989 | 0.990 | 0.992 | 0.993 |
| MAE, (mV) | 35.294 | 57.117 | 44.412 | 35.281 | 27.842 |
| Testing dataset |  |  |  |  |  |
| RMSE, (mV) | 109.824 | 187.091 | 190.442 | 210.223 | 162.525 |
| R^2^ | 0.967 | 0.952 | 0.927 | 0.929 | 0.940 |
| MAE, (mV) | 66.933 | 112.307 | 106.993 | 111.108 | 89.963 |
| P-R segment |  |  |  |  |  |
| Training dataset |  |  |  |  |  |
| RMSE, (mV) | 4.176 | 3.851 | 4.084 | 3.954 | 3.843 |
| R^2^ | 0.989 | 0.994 | 0.994 | 0.992 | 0.992 |
| MAE, (mV) | 2.706 | 2.735 | 2.872 | 2.789 | 2.572 |
| Testing dataset |  |  |  |  |  |
| RMSE, (mV) | 8.380 | 9.277 | 16.676 | 17.136 | 18.813 |
| R^2^ | 0.956 | 0.984 | 0.936 | 0.932 | 0.911 |
| MAE, (mV) | 5.068 | 6.002 | 7.651 | 7.979 | 7.947 |
| ST-T segment |  |  |  |  |  |
| Training dataset |  |  |  |  |  |
| RMSE, (mV) | 4.336 | 7.408 | 6.901 | 6.820 | 5.756 |
| R^2^ | 0.999 | 0.999 | 0.999 | 0.999 | 0.999 |
| MAE, (mV) | 2.774 | 4.850 | 4.431 | 4.365 | 3.628 |
| Testing dataset |  |  |  |  |  |
| RMSE, (mV) | 7.845 | 18.056 | 16.201 | 13.689 | 11.060 |
| R^2^ | 0.997 | 0.996 | 0.996 | 0.997 | 0.996 |
| MAE, (mV) | 5.590 | 12.431 | 10.703 | 9.232 | 7.739 |
| Approach 3 (XGBoost model) | |  |  |  |  |
| QRS complex |  |  |  |  |  |
| Training dataset |  |  |  |  |  |
| RMSE, (mV) | 7.292 | 6.490 | 26.105 | 5.107 | 10.111 |
| R^2^ | 1.000 | 1.000 | 0.998 | 1.000 | 1.000 |
| MAE, (mV) | 5.652 | 4.781 | 19.118 | 3.700 | 7.806 |
| Testing dataset |  |  |  |  |  |
| RMSE, (mV) | 99.376 | 179.604 | 179.926 | 193.256 | 149.708 |
| R^2^ | 0.973 | 0.953 | 0.932 | 0.939 | 0.949 |
| MAE, (mV) | 63.356 | 109.643 | 105.228 | 105.888 | 85.719 |
| P-R segment |  |  |  |  |  |
| Training dataset |  |  |  |  |  |
| RMSE, (mV) | 4.013 | 3.640 | 2.305 | 6.310 | 5.878 |
| R^2^ | 0.987 | 0.994 | 0.998 | 0.978 | 0.978 |
| MAE, (mV) | 2.903 | 2.768 | 1.659 | 4.823 | 4.393 |
| Testing dataset |  |  |  |  |  |
| RMSE, (mV) | 8.940 | 11.651 | 14.974 | 16.676 | 19.690 |
| R^2^ | 0.947 | 0.973 | 0.948 | 0.933 | 0.899 |
| MAE, (mV) | 6.108 | 7.452 | 7.878 | 9.187 | 9.100 |
| ST-T segment |  |  |  |  |  |
| Training dataset |  |  |  |  |  |
| RMSE, (mV) | 3.497 | 3.586 | 4.015 | 4.795 | 5.130 |
| R^2^ | 0.999 | 1.000 | 1.000 | 0.999 | 0.999 |
| MAE, (mV) | 2.589 | 2.574 | 2.897 | 3.560 | 3.777 |
| Testing dataset |  |  |  |  |  |
| RMSE, (mV) | 10.586 | 24.058 | 22.333 | 17.125 | 13.563 |
| R^2^ | 0.994 | 0.993 | 0.992 | 0.995 | 0.994 |
| MAE, (mV) | 7.634 | 16.945 | 15.320 | 12.480 | 9.942 |

MAE indicates mean absolute error; RMSE, root mean square error; R^2^, determination of coefficient (R-squared); XGBoost, eXtreme Gradient Boosting.

**Table S7. Five-fold cross-validation results of RF and XGBoost models**

|  |  | RF model | | | | XGBoost model | | | |
| --- | --- | --- | --- | --- | --- | --- | --- | --- | --- |
|  | Fold | RMSE (mV) | MAE (mV) | R^2^ | RMSE (mV) | | MAE (mV) | R^2^ |  |
| QRS Complex | |  |  |  |  | |  |  |  |
| Lead V1 | Fold 1 | 152.095 | 89.411 | 0.929 | 137.044 | | 94.571 | 0.942 |  |
|  | Fold 2 | 140.155 | 88.353 | 0.947 | 145.535 | | 99.986 | 0.943 |  |
|  | Fold 3 | 146.449 | 89.041 | 0.934 | 141.140 | | 95.432 | 0.939 |  |
|  | Fold 4 | 155.989 | 96.741 | 0.926 | 141.618 | | 99.915 | 0.939 |  |
|  | Fold 5 | 158.110 | 94.370 | 0.938 | 147.267 | | 98.541 | 0.946 |  |
|  | Mean ± SD | 150.56±7.314 | 91.583±3.741 | 0.935±0.008 | 142.521±4.01 | | 97.689±2.538 | 0.942±0.003 |  |
| Lead V2 | Fold 1 | 204.476 | 140.496 | 0.931 | 223.352 | | 159.828 | 0.922 |  |
|  | Fold 2 | 220.196 | 142.781 | 0.915 | 214.726 | | 156.688 | 0.918 |  |
|  | Fold 3 | 241.263 | 155.969 | 0.905 | 234.143 | | 167.540 | 0.908 |  |
|  | Fold 4 | 210.192 | 139.243 | 0.929 | 241.742 | | 166.437 | 0.905 |  |
|  | Fold 5 | 255.226 | 158.058 | 0.888 | 234.586 | | 166.006 | 0.906 |  |
|  | Mean ± SD | 226.271±21.41 | 147.309±8.979 | 0.914±0.018 | 229.71±10.644 | | 163.3±4.767 | 0.912±0.008 |  |
| Lead V3 | Fold 1 | 186.972 | 115.786 | 0.917 | 197.964 | | 140.553 | 0.915 |  |
|  | Fold 2 | 176.212 | 116.495 | 0.933 | 192.042 | | 135.410 | 0.914 |  |
|  | Fold 3 | 194.963 | 117.753 | 0.912 | 200.344 | | 134.218 | 0.902 |  |
|  | Fold 4 | 194.605 | 128.908 | 0.910 | 204.585 | | 138.061 | 0.902 |  |
|  | Fold 5 | 205.059 | 129.865 | 0.906 | 214.375 | | 143.529 | 0.898 |  |
|  | Mean ± SD | 191.562±10.72 | 121.762±7.004 | 0.915±0.01 | 201.862±8.335 | | 138.354±3.791 | 0.906±0.008 |  |
| Lead V4 | Fold 1 | 204.196 | 104.631 | 0.903 | 170.042 | | 111.639 | 0.932 |  |
|  | Fold 2 | 162.821 | 90.692 | 0.937 | 170.649 | | 106.006 | 0.925 |  |
|  | Fold 3 | 165.500 | 99.314 | 0.939 | 174.981 | | 111.204 | 0.929 |  |
|  | Fold 4 | 179.422 | 95.589 | 0.932 | 192.249 | | 116.495 | 0.915 |  |
|  | Fold 5 | 154.171 | 87.371 | 0.930 | 134.256 | | 92.609 | 0.957 |  |
|  | Mean ± SD | 173.222±19.549 | 95.519±6.84 | 0.928±0.015 | 168.435±21.129 | | 107.591±9.161 | 0.932±0.015 |  |
| Lead V5 | Fold 1 | 117.910 | 67.604 | 0.948 | 120.838 | | 77.218 | 0.949 |  |
|  | Fold 2 | 129.488 | 73.248 | 0.942 | 126.047 | | 80.542 | 0.950 |  |
|  | Fold 3 | 118.830 | 67.426 | 0.956 | 104.149 | | 70.739 | 0.963 |  |
|  | Fold 4 | 101.792 | 58.718 | 0.963 | 105.632 | | 66.631 | 0.963 |  |
|  | Fold 5 | 170.976 | 83.416 | 0.914 | 138.348 | | 78.525 | 0.937 |  |
|  | Mean ± SD | 127.799±26.085 | 70.082±9.085 | 0.945±0.019 | 119.003±14.375 | | 74.731±5.829 | 0.952±0.011 |  |
| ST-T Segment | |  |  |  |  | |  |  |  |
| Lead V1 | Fold 1 | 11.495 | 7.345 | 0.993 | 20.358 | | 15.155 | 0.978 |  |
|  | Fold 2 | 12.827 | 7.946 | 0.993 | 19.123 | | 14.404 | 0.981 |  |
|  | Fold 3 | 12.412 | 7.789 | 0.992 | 20.176 | | 14.916 | 0.979 |  |
|  | Fold 4 | 10.893 | 7.217 | 0.994 | 19.825 | | 14.786 | 0.981 |  |
|  | Fold 5 | 12.124 | 7.552 | 0.992 | 20.431 | | 15.066 | 0.981 |  |
|  | Mean ± SD | 11.95±0.764 | 7.57±0.302 | 0.993±0.001 | 19.983±0.534 | | 14.865±0.294 | 0.98±0.001 |  |
| Lead V2 | Fold 1 | 23.632 | 14.384 | 0.992 | 40.272 | | 29.296 | 0.976 |  |
|  | Fold 2 | 20.250 | 13.615 | 0.993 | 38.829 | | 28.799 | 0.979 |  |
|  | Fold 3 | 21.641 | 13.424 | 0.993 | 39.631 | | 29.001 | 0.976 |  |
|  | Fold 4 | 20.543 | 12.886 | 0.994 | 40.199 | | 29.167 | 0.974 |  |
|  | Fold 5 | 20.851 | 13.502 | 0.993 | 41.571 | | 30.135 | 0.972 |  |
|  | Mean ± SD | 21.383±1.36 | 13.562±0.538 | 0.993±0.001 | 40.1±1.005 | | 29.28±0.513 | 0.975±0.003 |  |
| Lead V3 | Fold 1 | 18.863 | 12.117 | 0.993 | 36.434 | | 26.401 | 0.974 |  |
|  | Fold 2 | 22.112 | 13.118 | 0.991 | 35.892 | | 25.586 | 0.974 |  |
|  | Fold 3 | 18.969 | 12.135 | 0.993 | 35.795 | | 26.245 | 0.978 |  |
|  | Fold 4 | 20.499 | 12.877 | 0.992 | 36.045 | | 26.803 | 0.974 |  |
|  | Fold 5 | 20.361 | 12.937 | 0.992 | 35.479 | | 26.166 | 0.977 |  |
|  | Mean ± SD | 20.161±1.329 | 12.637±0.475 | 0.992±0.001 | 35.929±0.35 | | 26.24±0.44 | 0.975±0.002 |  |
| Lead V4 | Fold 1 | 17.606 | 10.695 | 0.992 | 27.533 | | 20.215 | 0.982 |  |
|  | Fold 2 | 18.073 | 11.201 | 0.992 | 29.343 | | 20.862 | 0.980 |  |
|  | Fold 3 | 16.736 | 10.516 | 0.993 | 27.078 | | 19.938 | 0.983 |  |
|  | Fold 4 | 18.149 | 11.312 | 0.992 | 25.885 | | 19.252 | 0.983 |  |
|  | Fold 5 | 17.391 | 11.038 | 0.993 | 30.016 | | 20.948 | 0.977 |  |
|  | Mean ± SD | 17.591±0.573 | 10.952±0.338 | 0.992±0.001 | 27.971±1.689 | | 20.243±0.699 | 0.981±0.002 |  |
| Lead V5 | Fold 1 | 13.370 | 8.625 | 0.993 | 20.886 | | 15.244 | 0.982 |  |
|  | Fold 2 | 13.980 | 8.861 | 0.992 | 22.096 | | 15.777 | 0.981 |  |
|  | Fold 3 | 13.765 | 8.493 | 0.993 | 19.990 | | 14.367 | 0.985 |  |
|  | Fold 4 | 14.161 | 8.389 | 0.992 | 20.387 | | 14.543 | 0.983 |  |
|  | Fold 5 | 13.563 | 8.779 | 0.993 | 21.695 | | 15.812 | 0.982 |  |
|  | Mean ± SD | 13.768±0.316 | 8.63±0.195 | 0.993±0 | 21.011±0.879 | | 15.149±0.675 | 0.983±0.001 |  |
| P-R Segment | |  |  |  |  | |  |  |  |
| Lead V1 | Fold 1 | 10.877 | 7.167 | 0.905 | 13.452 | | 9.676 | 0.863 |  |
|  | Fold 2 | 10.110 | 6.979 | 0.915 | 13.541 | | 10.028 | 0.860 |  |
|  | Fold 3 | 11.422 | 7.709 | 0.905 | 13.903 | | 10.117 | 0.827 |  |
|  | Fold 4 | 10.817 | 7.136 | 0.909 | 12.661 | | 9.549 | 0.868 |  |
|  | Fold 5 | 10.745 | 7.113 | 0.906 | 13.515 | | 9.899 | 0.867 |  |
|  | Mean ± SD | 10.794±0.467 | 7.221±0.282 | 0.908±0.004 | 13.415±0.457 | | 9.854±0.238 | 0.857±0.017 |  |
| Lead V2 | Fold 1 | 10.005 | 7.078 | 0.958 | 11.921 | | 9.147 | 0.934 |  |
|  | Fold 2 | 9.731 | 7.018 | 0.956 | 12.308 | | 9.237 | 0.937 |  |
|  | Fold 3 | 10.037 | 7.285 | 0.954 | 12.475 | | 9.521 | 0.928 |  |
|  | Fold 4 | 11.021 | 7.624 | 0.946 | 13.159 | | 9.936 | 0.913 |  |
|  | Fold 5 | 10.144 | 7.424 | 0.950 | 11.492 | | 8.756 | 0.943 |  |
|  | Mean ± SD | 10.187±0.49 | 7.286±0.249 | 0.953±0.005 | 12.271±0.625 | | 9.32±0.44 | 0.931±0.011 |  |
| Lead V3 | Fold 1 | 12.405 | 8.081 | 0.943 | 12.253 | | 9.344 | 0.945 |  |
|  | Fold 2 | 10.166 | 7.405 | 0.961 | 11.757 | | 8.951 | 0.943 |  |
|  | Fold 3 | 12.136 | 7.990 | 0.946 | 12.903 | | 9.722 | 0.930 |  |
|  | Fold 4 | 11.261 | 7.911 | 0.947 | 12.354 | | 9.266 | 0.938 |  |
|  | Fold 5 | 9.955 | 7.161 | 0.959 | 12.843 | | 9.658 | 0.943 |  |
|  | Mean ± SD | 11.185±1.112 | 7.71±0.403 | 0.951±0.008 | 12.422±0.47 | | 9.388±0.313 | 0.94±0.006 |  |
| Lead V4 | Fold 1 | 10.547 | 7.416 | 0.937 | 11.100 | | 8.413 | 0.938 |  |
|  | Fold 2 | 9.982 | 7.377 | 0.940 | 12.658 | | 9.325 | 0.914 |  |
|  | Fold 3 | 9.821 | 7.036 | 0.950 | 11.754 | | 8.962 | 0.919 |  |
|  | Fold 4 | 10.966 | 7.459 | 0.937 | 10.805 | | 8.367 | 0.938 |  |
|  | Fold 5 | 9.675 | 7.172 | 0.951 | 11.071 | | 8.524 | 0.929 |  |
|  | Mean ± SD | 10.198±0.542 | 7.292±0.18 | 0.943±0.007 | 11.477±0.747 | | 8.718±0.413 | 0.927±0.011 |  |
| Lead V5 | Fold 1 | 9.737 | 6.277 | 0.940 | 9.138 | | 6.652 | 0.943 |  |
|  | Fold 2 | 8.023 | 5.942 | 0.956 | 9.458 | | 7.132 | 0.941 |  |
|  | Fold 3 | 8.564 | 6.032 | 0.952 | 9.774 | | 7.291 | 0.941 |  |
|  | Fold 4 | 9.410 | 5.701 | 0.948 | 10.705 | | 7.770 | 0.928 |  |
|  | Fold 5 | 8.701 | 6.172 | 0.950 | 9.651 | | 7.435 | 0.943 |  |
|  | Mean ± SD | 8.887±0.686 | 6.025±0.222 | 0.949±0.006 | 9.745±0.588 | | 7.256±0.411 | 0.939±0.006 |  |

MAE, mean absolute error; RMSE, root mean square error; R^2^, determination of coefficient (R-squared); SD, standard deviation; XGBoost, eXtreme Gradient Boosting.

**Table S8 Comparison of the model's diagnostic performance in training, testing, and external validation datasets**

|  | AUC (95% CI) | Specificity | Sensitivity | F1 Score |
| --- | --- | --- | --- | --- |
| Training dataset |  |  |  |  |
| Normal | 0.925 (0.897-0.953) | 0.950 | 0.900 | 0.886 |
| ST-segment elevation | 0.948 (0.910-0.985) | 0.983 | 0.912 | 0.897 |
| ST-segment depression | 0.909 (0.873-0.946) | 0.970 | 0.848 | 0.866 |
| Old myocardial infarction | 0.966 (0.933-0.998) | 1.000 | 0.931 | 0.964 |
| Bundle branch blocks | 0.992 (0.982-1.000) | 0.991 | 0.992 | 0.984 |
| Testing dataset |  |  |  |  |
| Normal | 0.970 (0.943-0.996) | 0.939 | 1.000 | 0.938 |
| ST-segment elevation | 1.000 (1.000-1.000) | 1.000 | 1.000 | 1.000 |
| ST-segment depression | 0.700 (0.540-0.860) | 1.000 | 0.400 | 0.571 |
| Old myocardial infarction | 1.000 (1.000-1.000) | 1.000 | 1.000 | 1.000 |
| Bundle branch blocks | 0.994 (0.982-1.000) | 0.988 | 1.000 | 0.988 |
| External validation dataset |  |  |  |  |
| Normal | 0.835 (0.734-0.908) | 0.839 | 0.656 | 0.832 |
| ST-segment elevation | 0.825 (0.693-0.923) | 0.795 | 0.714 | 0.847 |
| ST-segment depression | 0.898 (0.799-0.957) | 0.739 | 0.880 | 0.829 |
| Old myocardial infarction | 0.867 (0.622-0.956) | 0.764 | 1.000 | 0.866 |
| Bundle branch blocks | 0.910 (0.714-0.953) | 0.759 | 0.909 | 0.857 |

AUC, Areas under the curve; CI, confidence interval.

**Table S9. Subgroup performance of the model's diagnostic performance in the external validation datasets**

| Subgroup | Sensitivity | Specificity | Accuracy | AUC (95% CI) |
| --- | --- | --- | --- | --- |
| Sex |  |  |  |  |
| Female, (*n* = 9) | 0.667 | 0.917 | 0.867 | 0.704 (0.455–0.914) |
| Male, (*n* = 85) | 0.847 | 0.962 | 0.939 | 0.875 (0.801–0.924) |
| Age |  |  |  |  |
| >50 years, (*n* = 60) | 0.850 | 0.963 | 0.940 | 0.875 (0.794–0.933) |
| ≤50 years, (*n* = 34) | 0.794 | 0.949 | 0.918 | 0.832 (0.704–0.919) |
| BMI |  |  |  |  |
| >24 kg/m^2^, (*n* = 48) | 0.938 | 0.984 | 0.975 | 0.973 (0.926–0.993) |
| ≤24 kg/m^2^, (*n* = 46) | 0.717 | 0.929 | 0.887 | 0.786 (0.674–0.868) |

Body mass index (BMI), areas under the curve (AUC), confidence interval (CI).


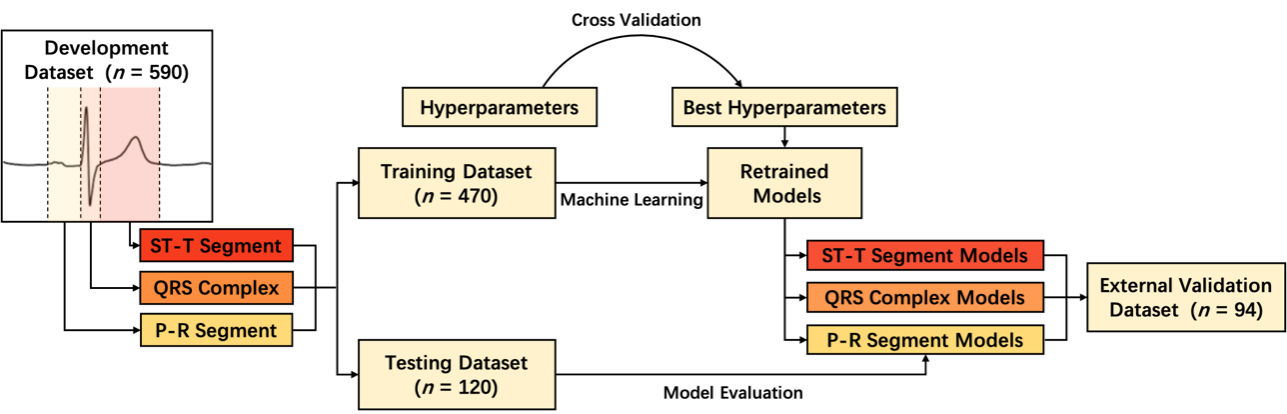


**Figure S1. The framework of the model development and validation.**

The electrocardiogram (ECG) data of development dataset (*n* = 590) were divided into P-R segment, QRS complex, and ST-T segment to establish models respectively. Then all the ECG data was divided into training and testing dataset. The Random Forest (RF) and eXtreme Gradient Boosting (XGBoost) methods were employed to establish P-R segment, QRS complex, and ST-T segment models for converting prone ECG lead voltage traces into standard ECGs. Parameters of each model were adjusted using cross-validation on the testing dataset. The performance of each model was evaluated in the testing group. The final models were externally validated using an external validation dataset (*n* = 94).

**Figure S2. The comparison of morphology and amplitude between the converted standard electrocardiograms (ECGs) using Approach 2 and the original ECGs.**

A. Comparison of QRS complex morphology between Dower and Uijen transformations converted standard ECGs and the original ECGs. Notably, lead V3 in the Dower transformation showed significantly better than the Uijen transformation (*P* < .001), while no significant differences were observed in other leads. B-E. Comparison of amplitude in each ECG waveform segment between Dower and Uijen transformations converted standard ECGs and the original ECGs. *, indicates statistically significant differences in amplitude compared to the original ECG.


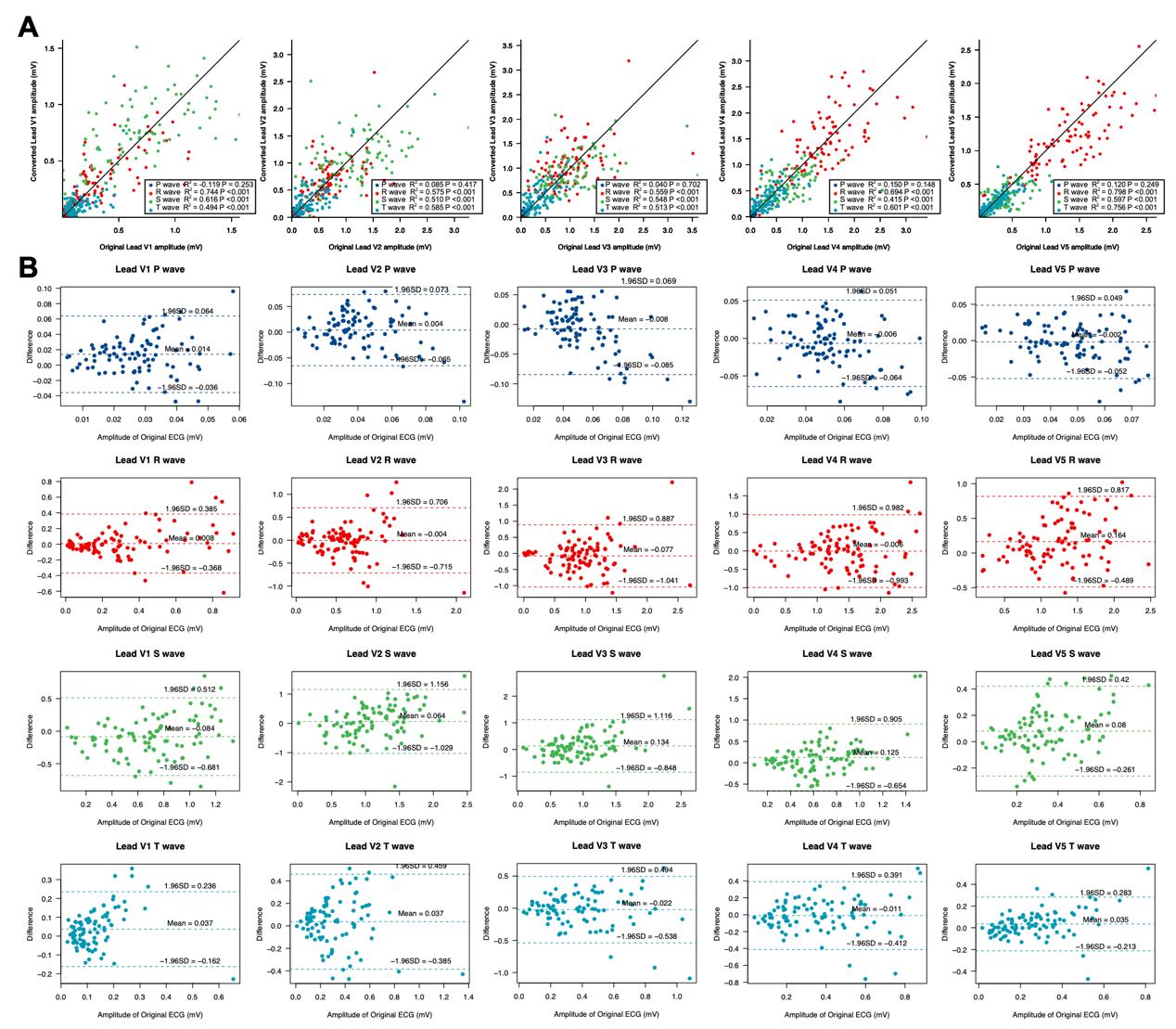


**Figure S3. Correlation of amplitudes and Bland-Altman analysis for each lead and waveform segment of electrocardiograms (ECGs) converted by the hybrid model in external validation set.**

A: Correlation plots comparing each lead and waveform segment of ECGs converted by the hybrid model in external validation set.

B: Bland-Altman analysis for each lead and waveform segment of ECGs converted by the hybrid model in external validation set. For each plot, the x-axis depicts the amplitude of each waveform segment in original ECG, and the y-axis plots the difference. The solid horizontal line depicts the overall mean difference, and the hashed lines depict the estimated 95% limits of agreement.

**Figure S4 Shapley additive explanation (SHAP) values for features the random forest model (Approach 3) to predict different segments (QRS complex, ST-T segment, P-R segment) of leads V1-V5.** A-E: QRS complex of leads V1-V5. F-J: ST-T segment of leads V1-V5. K-O: P-R segment of leads V1-V5. Feature importance is listed in descending order. Positive SHAP values (right side of the middle line) indicate higher likelihood of the outcome while negative SHAP values (left side of the middle line) indicate lower likelihood of the outcome. The feature sex was coded as follows: 0 = female, 1 = male.


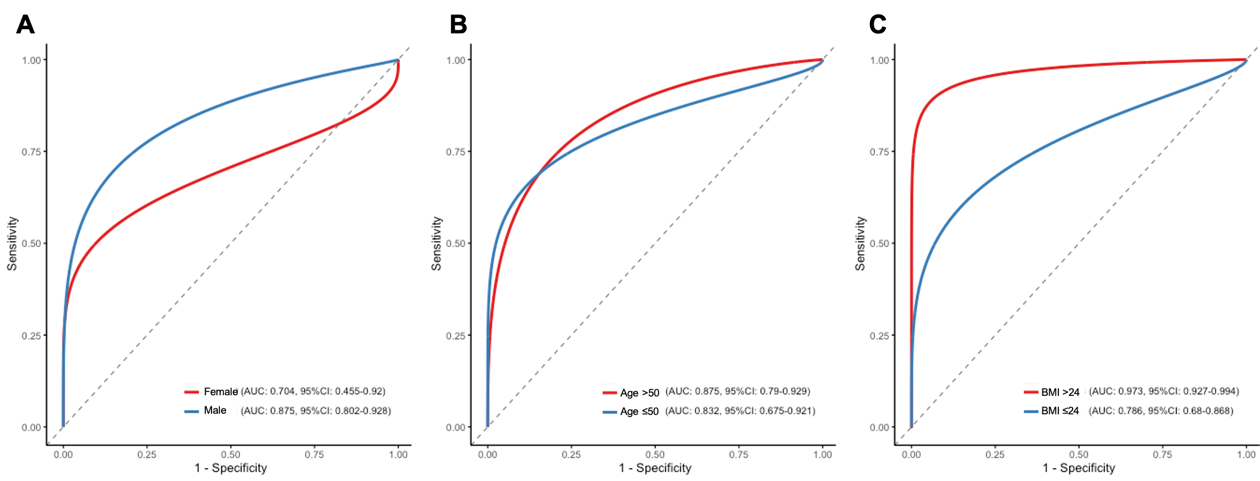


Figure S5 Areas under the receiver operating characteristic curve (AUROC) and 95% confidence interval (CI) of model diagnosis for each subgroup in the external validation dataset. A: Different gender subgroups; B: Different age subgroups (age >50 and ≤50 years); C: Different body mass index (BMI) subgroups (BMI >24 and ≤24 kg/m^2^)​​.
